# Geographically weighted linear combination test for gene-set analysis of a continuous spatial phenotype as applied to intratumor heterogeneity

**DOI:** 10.3389/fcell.2023.1065586

**Published:** 2023-03-09

**Authors:** Payam Amini, Morteza Hajihosseini, Saumyadipta Pyne, Irina Dinu

**Affiliations:** ^1^ Department of Biostatistics, School of Public Health, Iran University of Medical Sciences, Tehran, Iran; ^2^ School of Medicine, Keele University, Keele, Staffordshire, United Kingdom; ^3^ School of Public Health, University of Alberta, Edmonton, AB, Canada; ^4^ Stanford Department of Urology, Center for Academic Medicine, Palo Alto, CA, United States; ^5^ Health Analytics Network, Pittsburgh, PA, United States; ^6^ University of California, Santa Barbara, Santa Barbara, CA, United States

**Keywords:** intratumor heterogeneity, gene-set analysis, geographically weighted regression, linear combination test, Spatial single cell analysis, cancer-associated fibroblast

## Abstract

**Background:** The impact of gene-sets on a spatial phenotype is not necessarily uniform across different locations of cancer tissue. This study introduces a computational platform, GWLCT, for combining gene set analysis with spatial data modeling to provide a new statistical test for location-specific association of phenotypes and molecular pathways in spatial single-cell RNA-seq data collected from an input tumor sample.

**Methods:** The main advantage of GWLCT consists of an analysis beyond global significance, allowing the association between the gene-set and the phenotype to vary across the tumor space. At each location, the most significant linear combination is found using a geographically weighted shrunken covariance matrix and kernel function. Whether a fixed or adaptive bandwidth is determined based on a cross-validation cross procedure. Our proposed method is compared to the global version of linear combination test (LCT), bulk and random-forest based gene-set enrichment analyses using data created by the Visium Spatial Gene Expression technique on an invasive breast cancer tissue sample, as well as 144 different simulation scenarios.

**Results:** In an illustrative example, the new geographically weighted linear combination test, GWLCT, identifies the cancer hallmark gene-sets that are significantly associated at each location with the five spatially continuous phenotypic contexts in the tumors defined by different well-known markers of cancer-associated fibroblasts. Scan statistics revealed clustering in the number of significant gene-sets. A spatial heatmap of combined significance over all selected gene-sets is also produced. Extensive simulation studies demonstrate that our proposed approach outperforms other methods in the considered scenarios, especially when the spatial association increases.

**Conclusion:** Our proposed approach considers the spatial covariance of gene expression to detect the most significant gene-sets affecting a continuous phenotype. It reveals spatially detailed information in tissue space and can thus play a key role in understanding the contextual heterogeneity of cancer cells.

## Introduction

Globally, there were approximately 2.3 million new cases of and 685,000 deaths due to breast cancer (BC) in 2020 (Lei et al., 2021). BC is also the leading cause of cancer-related deaths among women (Beiki et al., 2012; World Health Organization, 2018) and the second leading cause of cancer deaths globally, worldwide (Beiki et al., 2012). The tumorigenesis involves uncontrolled growth of cells in breast tissues which can be either benign or malignant (Liu et al., 2013). Several studies on breast cancer patients have revealed the different anti- and pro-tumorigenic roles of the CAFs involved (Chang et al., 2012; Brechbuhl et al., 2017; Su et al., 2018).

Extensive studies over the past few decades have uncovered a variety of cell populations in tumors, thus leading to the active research area of intratumor heterogeneity (ITH) (Marusyk et al., 2020). In 2010, Hanahan and Weinberg noted that tumors exhibit an additional dimension of complexity through their “tumor microenvironment” that contributes to the acquisition of the so-called hallmark traits of cancer. ITH is attributed to genetic, epigenetic, and microenvironmental factors (McGranahan and Swanton, 2017; Marusyk et al., 2020) and associated with poor prognosis, therapeutic resistance and treatment failure leading to poor overall survival in cancer patients (Landau et al., 2013; Patel et al., 2014; Zhang et al., 2014; Jamal-Hanjani et al., 2015; Jamal-Hanjani et al., 2017). Indeed, the persistence of some of the drug-tolerant intratumor cell populations could be attributed to their high phenotypic plasticity (Flavahan et al., 2017).

Interestingly, hierarchies of differentiation also exist among normal cells in healthy tissues, but the populations of tumor cells display far greater cell-to-cell variability and the resulting phenotypic instability (Landau et al., 2014; Jenkinson et al., 2017). Such ITH could be attributed to genetic causes ranging from aneuploidy to other factors such as complex contextual signals in the highly aberrant tumor microenvironments, or even global alterations in cancer cell epigenomes (Senovilla et al., 2012). ITH also involves immune cell infiltration, which is important to immunotherapies. Tumor antigen diversity could be determined by the T Cell clonality in the different regions of the same tumor (Senovilla et al., 2012). Studies have shown spatially complex interactions between tumor microenvironments and the patient’s immune system (Hanahan and Weinberg, 2011; Vogelstein and Kinzler, 2015).

While heterogeneous cell types are prevalent within the tumor microenvironment, some of which may account for cancer development and progression, it also contains different non-malignant components, including the cancer-associated fibroblasts (CAFs) (Pietras and Östman, 2010; Cortez et al., 2014; Kalluri, 2016). Although the origin and activation mechanism of CAFs remains an area of active research (Anderberg and Pietras, 2009; Shiga et al., 2015; LeBleu and Kalluri, 2018; Chen and Song, 2019), studies have attributed the processes of formation and derivation of CAFs to various precursor cells (Anderberg and Pietras, 2009; Shiga et al., 2015; LeBleu and Kalluri, 2018; Chen and Song, 2019), which may be the source of the well-known heterogeneity among the CAFs (Du and Che, 2017; Öhlund et al., 2017; Costa et al., 2018; Raz et al., 2018; Lee et al., 2020). Indeed, in certain tumors, such as in the breast, in which the prevalence of CAFs could be as high as 80%, they can play both anti-as well as pro-tumorigenic roles (Chang et al., 2012; Brechbuhl et al., 2017; Su et al., 2018). Importantly, CAFs can facilitate drug resistance dynamically by altering the cell-matrix interactions that control the outer layer of cells’ sensitivity to apoptosis, producing proteins that control cell survival and proliferation, assisting with cell-cell communications, and activating epigenetic plasticity in neighboring cells (Cuiffo and Karnoub, 2012; Junttila and De Sauvage, 2013). CAF-targeted treatments can have dual effects depending on the target and the tissue under consideration (Özdemir et al., 2014; Koliaraki et al., 2015; Wagner, 2016). For instance, spatial proximity to CAFs has been shown to impact molecular features and therapeutic sensitivity of breast cancer cells influencing clinical outcomes (Marusyk et al., 2016).

In recent years, higher resolution, tissue-specific gene expression analysis is made possible by using new platforms such as single-cell RNA sequencing (scRNA-seq), which has rapidly evolved as a powerful and popular tool (Kalisky et al., 2018; Sun et al., 2021). Unlike previous transcriptomic studies that assayed a “bulk” sample, scRNA-seq data can provide a detailed characterization of each tumor. Indeed, the Human Tumor Atlas Network [https://humantumoratlas.org] is increasingly enriched with data on human cancers based on scRNA-seq assays. The high-resolution transcriptomic platform has led to several scRNA-seq studies of the composition of CAFs in different stages of cancer (Bernardo and Fibbe, 2013; Li et al., 2017; Puram et al., 2017; Lambrechts et al., 2018; Elyada et al., 2019; Hosein et al., 2019; Davidson et al., 2020; Dominguez et al., 2020; Friedman et al., 2020). For focused understanding of the heterogeneous expressions of genes, different sites of the same tumor were analyzed with multiregional RNA sequencing for different cancers (Gerlinger et al., 2012; Zhang et al., 2014; Yates et al., 2015; Thrane et al., 2018).

Despite the advancements and efficacy of scRNA-seq, the lack of spatial information in scRNA-seq analysis is a significant shortcoming for typical scRNA-seq methods to capture cellular heterogeneity. For a tumor sample, the presence of spatial contexts might play a major role which could be combined with scRNA-seq data with the explicit aim to capture microenvironmental heterogeneity. Spatial cell-to-cell communication in a given tissue image can be recovered from a spatial scRNA sequencing data *via* computational spatial re-mapping (Teves and Won, 2020). Alternatively, integration of high-resolution gene expression data with spatial coordinates can resolve such experimental shortcomings (Eng et al., 2019). While imaging the transcriptome *in situ* with high accuracy has been a major challenge in single-cell biology, development of high-throughput platforms for sequential fluorescence *in situ* hybridization such as RNA seqFISH+ and algorithms such as CELESTA can identify cell populations and their spatial organization in intact tissues (Zhang et al., 2014; Eng et al., 2019). Towards this, many recent efforts have developed methods to analyze spatial information in single-cell studies (Lee et al., 2014; McKenna et al., 2016; Shah et al., 2016; Frieda et al., 2017; Alemany et al., 2018; Codeluppi et al., 2018; Raj et al., 2018; Spanjaard et al., 2018; Wang et al., 2018).

High-throughput transcriptomic data are useful not only for identifying genes that are differentially expressed, but also to test for co-regulation of multiple genes, i.e., a gene-set, based on existing empirical knowledge of biological pathways and gene signatures, e.g., the well-known hallmarks of cancer. In this direction, several methods for gene-set analysis (GSA) were introduced by (Goeman et al., 2004; Mansmann and Meister, 2005; Subramanian et al., 2005; Kong et al., 2006; Dinu et al., 2007; Efron and Tibshirani, 2007). Since the genes within such gene-sets share a common biological function, considering the correlations within each set is a key aspect of a useful GSA method. However, it was shown by (Tsai and Chen, 2009) that the above GSA methods were affected by large type II errors.

An important limitation of many GSA methods is that they can only accommodate binary outcomes, such as disease *versus* control. Our method, Linear Combination Test (LCT) is a GSA method that was designed to address these limitations by taking into account correlations across genes and outcomes, and dealing with binary, univariate or multivariate continuous outcomes, measured either at a single point in time or at multiple time points, and therefore, allow us to analyze a wider range of studies involving complex study designs (Wang et al., 2014). Studies have shown that LCT can overcome difficulties such as small sample size, large gene-sets, and can accommodate correlations across gene-sets, time points, and multiple correlated continuous phenotypes (Dinu et al., 2013). Thus, while a specific gene may not show consistent expression across individual cells, LCT is more likely than traditional approaches to detect the regulation of a functional process or biological pathway associated with the intercellular diversity of outcomes in a single cell level experiment.

Recently, we have extended LCT beyond any other “bulk” GSA method for application to single cell experiments (Dinu et al., 2021). However, GSA is considerably more complicated in the presence of spatial information since the analyzed gene-sets need not have a uniform impact over the entire area of a spatially continuous phenotype. In fact, the significance of association between a selected gene-set and a particular phenotypic context at various microenvironmental neighborhoods could be different. Yet, variable as they may be, since spatial effects are generally continuous in nature, proximity may determine more correlated associations than those across distant locations within the same tumor space. Notably, this alludes to Tobler’s First Law of Geography, which states that “everything is related to everything else, but near things are more related than distant things.” Traditional testing of such relationships involves global or “aspatial” regression, with the implicit assumption that the impact of the genes in a gene-set (covariates) on the phenotype (spatial outcome) is constant across the tumor space (study area). In the presence of ITH, such stationarity assumption is unlikely to be valid. Geographically weighted regression (GWR) is a well-known method (Brunsdon et al., 1996) that avoids this problem by performing the regression within local windows and each observation is weighted according to its proximity to the center of the window. Adaptive kernel bandwidths allow for heterogeneity among densities of gene expression over the windows in different parts of the study region. Local regression coefficients and associated statistics are mapped to visualize how the explanatory power of a gene-set on the associated phenotypes changes spatially.

In the present study, we combined gene-set analysis of LCT with the local spatial modeling of GWR with the aim to develop geographically weighted LCT (GWLCT) as a statistical test. We demonstrated it on spatial scRNA-seq data from a real breast tumor sample and obtained key insights into its molecular heterogeneity across different spatially continuous phenotypic contexts defined by five well-known markers of CAFs. We note that GWLCT has several distinct advantages. While the popular GSA methods are aspatial and use only bulk gene expression data, GWLCT is developed for spatial single cell gene expression data. The geographical weighting scheme allows nearby neighborhoods to contribute more to each local model, and the regions with significant association of a selected gene-set and a corresponding phenotype are detected using scan statistics on the local test scores and illustrated as maps. At each location, the combined significance of such associations for the selected gene-sets is computed and visualized with a spatial heatmap. We also present new 3D interactive tools for insightful visualization of the tumor space. In the next section, we describe the data and methods, followed by the results of real tumor data analysis and simulations of different association scenarios using GWLCT, and end with discussion, including future work.

## Materials and methods

### Data

Data for spatial transcriptomics were downloaded from the 10x Genomics website (https://www.10xgenomics.com/). In brief, the data were created using the Visium Spatial Gene Expression technique on an invasive breast cancer tissue sample that is expressing the Estrogen Receptor (ER), Progesterone Receptor (PR), and Human Epidermal Growth Factor Receptor (HER) negative. Illumina NovaSeq 6000 was used to generate the RNA sequencing data, which had a sequencing depth of 72,436 mean reads per cell. The downloaded dataset was filtered for average gene expression values greater than 1, and the resulting data matrix had 1,981 rows (genes) and 4,325 columns (single cells). The zero counts were substituted as part of the RNAseq data preparation with a relatively small random jitter about zero that would have the least impact on the remaining gene expression values. Using the bestNormalize package in the R programming language, we used a 10-fold cross-validation based data transformation strategy to normalize each gene’s expression across samples (Peterson and Peterson, 2020).

### Gene-sets

We downloaded from the Molecular Signatures Database (MSigDB) candidate gene-sets that represent commonly known “hallmarks” of cancer (Liberzon et al., 2011). To ensure their relevance and non-redundancy, we selected 8 hallmark gene-sets with at least 25% overlap with the expressed genes (see above text on preprocessing) but mutual gene-set overlap of less than 10%. The selected hallmark gene-sets are: Epithelial Mesenchymal Transition (EMT, size = 81) (Sun et al., 2020), Angiogenesis (size = 12) (Madu et al., 2020), DNA Repair (DNA_Rep, size = 42) (Paluch-Shimon and Evron, 2019), Pi3k AKT MTOR Signaling (Pi3k, size = 28) (Dong et al., 2021), Fatty Acid Metabolism (FAM, size = 41) (Xu et al., 2021), P53 Pathway (P53, size = 50) (Gasco et al., 2002), Estrogen Response Early (ERE, size = 63) (Oshi et al., 2020), and Estrogen Response Late (ERL, size = 62) (Takeshita et al., 2021).

### CAF markers

A selected set of five CAF phenotypes, which were represented by the expression of the corresponding marker genes (the respective phenotypes are noted in parentheses): *CXCL12* (CAF-S1), *FBLN1* (mCAFs), *C3* (inflammatory CAFs), S100A4 (sCAFs), and *COL11A1*, which is a fibroblast-specific “remarkable biomarker” that codes for collagen 11-α1 and shows expression gain in CAFs (Vázquez-Villa et al., 2015). For details on the CAF markers, see reviews, e.g., (Gascard and Tlsty, 2016; Lee et al., 2020).

### Statistical analysis

Similar to our previously developed GSA methods, GWLCT is motivated by a research gap, more precisely, the need for a statistical method taking into account spatial correlations across genes. The main goal of GSA methods is to efficiently screen large catalogues of *a priori* defined sets of genes sharing common biological functions, easily accessible to GSA users. GSA methods are testing for associations of such sets with a phenotype. To the best of our knowledge, there are no such methods developed for situations where gene measurements at spatial proximity could exhibit higher correlations. What is popularly known as Tobler’s “first law of geography” states that “everything is related to everything else, but near things are more related than distant things.” Based on this fundamental concept, which we borrowed from spatial data analysis, we provide below statistical derivations of an extension of LCT to geographically weighted spatial omic data.

Consider gene expression data of “ g ” gene variables ( $X_{1},X_{2},X_{3},\ldots ,X_{g}$ ), “ L ” cells (points) and “ K ” sets of genes at locations given by 2-dimensional Cartesian coordinates. The LCT approach assumes a null hypothesis in which there is no association between a linear combination of $X_{1},X_{2},X_{3},\ldots ,X_{g}$ with the phenotype (Dinu et al., 2013). For a local point $\left(u_{l},v_{l}\right)$ , we can define a univariate regression as:

$$Y_{\left(u_{l},v_{l}\right)}={\alpha_{0}}_{\left(u_{i},v_{i}\right)}+{\alpha_{1}}_{\left(u_{l},v_{l}\right)}{\beta_{1}X}_{1\left(u_{l},v_{l}\right)}+\ldots ++{\alpha_{g}}_{\left(u_{l},v_{l}\right)}{\beta_{g}X}_{g\left(u_{l},v_{l}\right)}+\varepsilon_{\left(u_{l},v_{l}\right)}$$

where

$${Z\left(\beta \right)}_{\left(u_{l},v_{l}\right)}={\alpha_{0}}_{\left(u_{l},v_{l}\right)}+{\alpha_{1}}_{\left(u_{l},v_{l}\right)}{\beta_{1}X}_{1\left(u_{l},v_{l}\right)}+\ldots ++{\alpha_{g}}_{\left(u_{l},v_{l}\right)}{\beta_{g}X}_{g\left(u_{l},v_{l}\right)}$$

is the linear combination of $X_{1},X_{2},X_{3},\ldots ,X_{g}$ and $\varepsilon_{\left(u_{l},v_{l}\right)}∼N\left(0,\sigma^{2}\right)$ . For each location in the dataset, we can find the most significant linear combination as follows:

$$\beta_{\left(u_{l},v_{l}\right)}^{*}=\mathrm{Max}\left[\rho \left(Y_{\left(u_{l},v_{l}\right)},Z_{\left(u_{l},v_{l}\right)}\right)\right]=\mathrm{Max}\left[\frac{\beta_{\left(u_{l},v_{l}\right)}^{T}{SCov\left(Y,X\right)}_{\left(u_{l},v_{l}\right)}\beta_{\left(u_{l},v_{l}\right)}^{T}}{\beta_{\left(u_{l},v_{l}\right)}^{T}{SCov\left(X,X\right)}_{\left(u_{l},v_{l}\right)}\beta_{\left(u_{l},v_{l}\right)}^{T}}\right]$$

where SCov represents the weighted shrunken covariance matrix for each calibration location. The weights are generated using a bisquare kernel function, based on the Euclidean distance between two points l and $l^{\prime }$

$$d_{ll^{\prime }}=\sqrt{{\left({Longitude}_{l}-{Longitude}_{l^{\prime }}\right)}^{2}+{\left({Latitude}_{l}-{Latitude}_{l^{\prime }}\right)}^{2}}$$

and bandwidth $h_{l}$ , which determines the radius around the point l . Here, the optimal bandwidth is calculated using cross validation (CV) based on the sum of squared errors at each cell point and set of genes:

$$CV=\overset{K}{\underset{k=1}{\sum }}\overset{C}{\underset{i=1}{\sum }}\overset{C}{\underset{j=1}{\sum }}{\left(Y_{\left(u_{i}.v_{i}\right).k}-{\hat{Y}}_{\left(u_{j\neq i}.v_{j\neq i}\right).k}\right)}^{2}$$

The bandwidth with the least measure of CV is used for localization. Weighting functions of bisquare and tricube type kernels are used to take the weighted location at l against another location $l^{\prime }$ into account. The bisquare kernel weighting function is defined as:

$$w_{ll^{\prime }}\left(u_{i}.v_{i}\right)=\{{\left[1-{\left(\frac{d_{ll^{\prime }}}{h_{l}}\right)}^{2}\right]}^{2};\;d_{ll^{\prime }}<h_{l}\;0\;;d_{ll^{\prime }}\geq h_{l}$$

and the tricube kernel weighting function as:

$$w_{ll^{\prime }}\left(u_{i}.v_{i}\right)=\{{\left[1-{\left(\frac{d_{ll^{\prime }}}{h_{l}}\right)}^{3}\right]}^{3};\;d_{ll^{\prime }}<h_{l}\;0\;;d_{ll^{\prime }}\geq h_{l}.$$

For the weighting functions, the bandwidth can be determined either beforehand (fixed distance) or as the distance between the point *l* and its nearest neighbor (adaptive), which is predetermined as well.

The shrunken covariance matrix of the gene expression data in the $l^{th}$ cell and around the estimated bandwidth ( h ) can be written as:

$${SCov\left(X.X\right)}_{\left(u_{l}.v_{l}\right)}=\left[\begin{array}{ccc} \frac{1}{L-1}\overset{L}{\underset{l^{\prime }=1}{\sum }}w_{ll^{\prime }}\left(x_{1ll^{\prime }}-{\bar{x}}_{1}\right)\left(x_{1ll^{\prime }}-{\bar{x}}_{1}\right) & \cdots & \frac{1}{L-1}\overset{L}{\underset{l^{\prime }=1}{\sum }}w_{ll^{\prime }}\left(x_{1ll^{\prime }}-{\bar{x}}_{1}\right)\left(x_{gll^{\prime }}-{\bar{x}}_{g}\right)\\ \vdots & \ddots & \vdots \\ \frac{1}{L-1}\overset{L}{\underset{l^{\prime }=1}{\sum }}w_{ll^{\prime }}\left(x_{gll^{\prime }}-{\bar{x}}_{g}\right)\left(x_{1ll^{\prime }}-{\bar{x}}_{1}\right) & \cdots & \frac{1}{L-1}\overset{L}{\underset{l^{\prime }=1}{\sum }}w_{ll^{\prime }}\left(x_{gll^{\prime }}-{\bar{x}}_{g}\right)\left(x_{gll^{\prime }}-{\bar{x}}_{g}\right) \end{array}\right]$$

$${SCov\left(Y.X\right)}_{\left(u_{l}.v_{l}\right)}=\left[\begin{array}{ccc} {\left[\frac{1}{L-1}\overset{L}{\underset{l^{\prime }=1}{\sum }}w_{ll^{\prime }}\left(y_{ll^{\prime }}-\bar{y}\right)\left(x_{1ll^{\prime }}-{\bar{x}}_{1}\right)\right]}^{2} & \cdots & \frac{1}{{\left(L-1\right)}^{2}}\overset{L}{\underset{l^{\prime }=1}{\sum }}w_{ll^{\prime }}\left(y_{ll^{\prime }}-\bar{y}\right)\left(x_{1ll^{\prime }}-{\bar{x}}_{1}\right)\overset{L}{\underset{l^{\prime }=1}{\sum }}w_{ll^{\prime }}\left(y_{ll^{\prime }}-\bar{y}\right)\left(x_{gll^{\prime }}-{\bar{x}}_{g}\right)\\ \vdots & \ddots & \vdots \\ \frac{1}{{\left(L-1\right)}^{2}}\overset{L}{\underset{l^{\prime }=1}{\sum }}w_{ll^{\prime }}\left(y_{ll^{\prime }}-\bar{y}\right)\left(x_{gll^{\prime }}-{\bar{x}}_{g}\right)\overset{L}{\underset{l^{\prime }=1}{\sum }}w_{ll^{\prime }}\left(y_{ll^{\prime }}-\bar{y}\right)\left(x_{1ll^{\prime }}-{\bar{x}}_{1}\right) & \cdots & {\left[\frac{1}{L-1}\overset{L}{\underset{l^{\prime }=1}{\sum }}w_{ll^{\prime }}\left(y_{ll^{\prime }}-\bar{y}\right)\left(x_{gll^{\prime }}-{\bar{x}}_{g}\right)\right]}^{2} \end{array}\right]$$

Using the weighted shrunken covariance matrices, the most significant linear combination at location $\left(u_{l},v_{l}\right)$ can be determined as the maximum Eigenvector of ${SCov\left(Y,X\right)}_{\left(u_{l},v_{l}\right)}{{SCov\left(X,X\right)}_{\left(u_{l},v_{l}\right)}}^{-1}$

### Combined significance mapping

We used the Fisher’s sum of log of independent *p*-values method to calculate the combined significance ( CS ) of association of the K gene-sets (e.g., 8 hallmarks in the present example) with a fixed phenotype at each location. The sum $X^{2}=\left(-2\right)\times \sum_{k=1}^{K}\log p_{k}$ follows a chi-square distribution with 2K degrees of freedom, which yields a combined *p*-value CP corresponding to $X^{2}$ at a given location. Thus, the combined significance is computed as $CS=\left(-1\right)\times \log CP$ and plotted as a spatial heatmap.

### Spatial cluster detection

Based on the count of significant gene-sets as determined by GWLCT at a given location, spatial clusters are detected and mapped for the user. For this purpose, assuming a Poisson distribution of such counts over a grid of points placed on the tumor space, scan statistics are computed with Openshaw’s Geographical Analysis Machine (GAM) (Openshaw et al., 1987) function as implemented in the R package DCluster.

### Comparative analysis

A comparative analysis against popular aspatial GSA methods should help the reader understand the relevance of GWLCT extension to the GSA literature. In addition to GWLCT, the global LCT, GSEA, and a Random Forest based GSEA (RF-GSEA) (Chien et al., 2014) were also performed to identify the global gene-sets associated with the outcome. In the domain of regression, the random forest based technique is used when the outcome of concern is a continuous phenotype. The GSEA ignores the continuous phenotype and checks if the gene-sets show statistical difference between biological states. The RF-GSEA combines bootstrap and classification tree to find the proportion of explained variance of a continuous phenotype for a specific gene-set. The small sample size issue has been previously considered in these methods so that variable selection is conducted only from a small random subset of the variables. Moreover, the RF-GSEA is able to accommodate a continuous phenotype when the associations between genesets and phenotypes are non-linear and contain complicated high-order interaction effects (Chien et al., 2014). Next, we give details of the simulation studies, including our approach to generate low and high spatial correlations among genes’ expressions. This is a key aspect in observing and understanding advantages of GWLCT over aspatial methods in our study.

### Simulation study

Several scenarios were designed to find the impact of simulation components such as bandwidth, number of coordinate points, number of genes, genes spatial association, phenotype-genes spatial association, and gene set probability on the statistical power of the methods. Similar to our previous simulations studies for LCT and its extensions (Wang et al., 2014), we generated a random subset of genes. The first step of the simulation was designed by making assumptions for the spatial distribution of the genes and phenotype. To do so, we used spatial and spatio-temporal geostatistical modeling, prediction and simulation function in R software called “gstat”. This function creates an R object with the necessary fields for univariate or multivariate geostatistical prediction, its conditional or unconditional Gaussian, or indicator simulation equivalents (Pebesma, 2004). Values were set for the variogram model components as 10 for the partial sill, 3 for the range parameter, 10 for the nugget, and 30 for the number of nearest observations that are used for the kriging simulation. Moreover, a Gaussian model was assumed for the distribution of the gene expressions and phenotypes.

In the next step, the GWLCT components were defined. For the gene-set matrix, a binomial distribution was used to generate a membership indicator matrix in which the proportions of genes belonging to the gene-sets were characterized using the probability parameter (Low = 0.3, and High = 0.9). Three different values for the spatial covariance were considered to imply the spatial association among genes’ expressions. The higher the spatial covariance, the lower the spatial association. Thus, a variance of 50 was considered as high which gives a corresponding low spatial association, a variance of 5 a moderate spatial association, and a variance of 0.1 a high spatial association.

As well, the spatial association between the continuous phenotype and the gene expression data was taken into account by a spatially and normally distributed phenotype generated from the gene expression data with the same parameters as for the spatial association among genes’ expressions. High and low levels for the radius/bandwidth around each location for the local analyses were 20 and 6, respectively. The number of coordinate points was 10 by 10 for low, and 100 by 100 for high. Finally, the two levels of low and high were defined for the total number of genes as 100 and 1000 respectively.

The above simulation components resulted in 144 scenarios in which an adaptive bandwidth kernel with the bisquare weighting function was used. The number of permutations was fixed and considered as 500, and the threshold of significance was assumed as 0.05. The methods were compared based on their statistical power. An average of statistical powers across the locations was computed and used as the performance measure for GWLCT.

R programming software version 4.1.1 is used for data analysis and packages such as corpcor (Schafer et al., 2017), qvcalc (Firth and Firth, 2020), stringr (Wickham, 2010), and plotly (Sievert, 2020).

## Results

**FIGURE 1:**
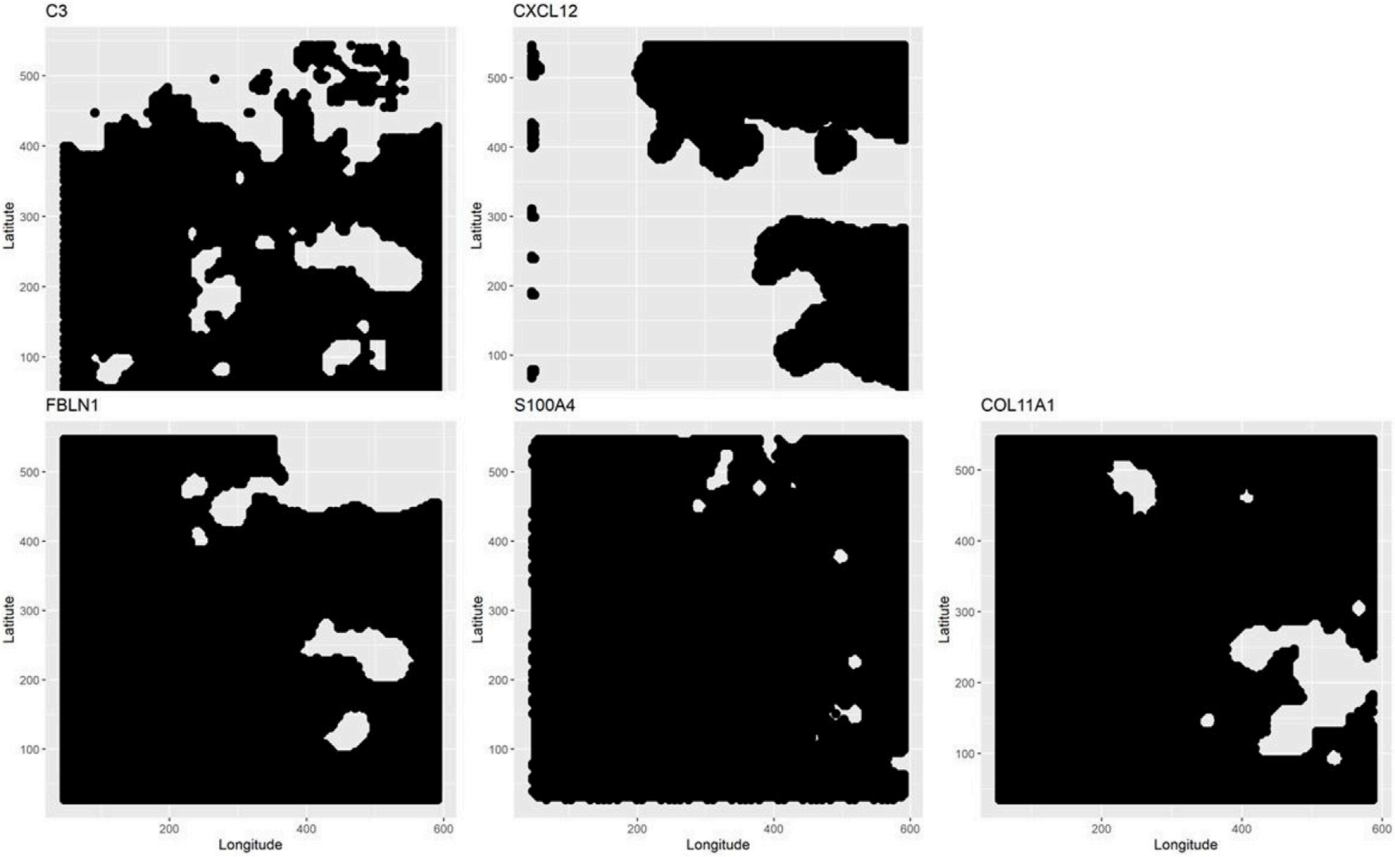
Scan statistics are used to detect and demarcate the putative clusters of cells which are enriched with selected gene-sets. The spatial clusters are defined on the tumor space by a scan statistic based on a Poisson distribution. At the locations where the scan statistics exceed a pre-specified threshold, the detected clusters for each phenotype are shown in white.

The three aspatial methods, LCT, RF-GSEA, and GSEA, were used to identify the “hallmark” cancer gene-sets that are significantly associated with five spatially continuous CAF phenotypes represented by their known markers C3, COL11A1, CXCL12, FBLN1, and S100A4 in the single cell breast cancer spatial transcriptomic data. The three aspatial methods, the phenotype, the size of each gene-set, *p*-value of the test, and the corresponding q-value are shown in Table 1. We note that such global *p*-values cannot be obtained from our proposed GWLCT, as this method is spatial in nature and assesses significance at every coordinate point rather than an overall significance measure. Using GSEA, the only significant gene-set was P53 (*p*-value = 0.009, q-value = 0.010). The results of LCT and RF-GSEA revealed that all the gene-sets are strongly associated with the five continuous phenotypes with *p*-value and q-value less than 0.001. This was expected since the candidate gene-sets represent commonly known “hallmarks” of cancer (Liberzon et al., 2011). The breast cancer data analysis interpretation resulting from the three aspatial methods considered is limited to the global significance values. This is an important limitation of aspatial methods. For the remainder of this section, we emphasize the advantages of GWLCT results interpretation in the context of spatial data analysis. Since the main advantage of GWLCT consists of an analysis beyond global significance, we study specifically at locations across the tumor space. Scan statistics provide a well-established computational method for detecting spatial clusters based on point count data. We computed scan statistics to detect the putative clusters of spatial regulation based on the number of cells with significantly enriched gene-sets occurring at locations where such counts exceeded what may be expected from an underlying Poisson distribution defined over the tumor space. The clusters are demarcated as white regions in Figure 1. Notably, the 3D plots, which are instrumental in showcasing the advantages of GWLCT over existing aspatial GSA methods, are presented here as well as at the following links on GitHub: https://mortezahaji.github.io/GWLCT-Project/. By clicking on any point in the plot, one can find the coordinate of the cell, corresponding number and name of significant gene-sets at three different levels: Low, Moderate, and High expressions of the selected CAF marker gene.

**TABLE 1:** An evaluation of cancer hallmark gene-sets associated with five continuous phenotypes C3, COL11A1, CXCL12, FBLN1, and S100A4 using the aspatial methods including GSEA, LCT, and RF-GSEA on a single cell breast cancer study.

Method	Phenotype	Gene-set name	Gene-set size	*p*-value	Q-value
GSEA (No phenotype specified)	Not Applicable	EMT	81	0.504	0.648
		ANGIOGENESIS	12	0.227	0.486
		DNA_Rep	41	0.425	0.763
		PI3K	28	0.059	0.155
		FAM	40	0.623	0.648
		P53	50	0.009	0.010
		ERE	64	0.178	0.486
		ERL	62	0.297	0.486
Global LCT	C3, COL11A1, CXCL12, FBLN1, S100A4	EMT	81	<0.001	<0.001
		ANGIOGENESIS	12	<0.001	<0.001
		DNA_Rep	41	<0.001	<0.001
		PI3K	28	<0.001	<0.001
		FAM	40	<0.001	<0.001
		P53	50	<0.001	<0.001
		ERE	64	<0.001	<0.001
		ERL	62	<0.001	<0.001
RF-GSEA	C3, COL11A1, CXCL12, FBLN1, S100A4	EMT	81	<0.001	<0.001
		ANGIOGENESIS	12	<0.001	<0.001
		DNA_Rep	41	<0.001	<0.001
		PI3K	28	<0.001	<0.001
		FAM	40	<0.001	<0.001
		P53	50	<0.001	<0.001
		ERE	64	<0.001	<0.001
		ERL	62	<0.001	<0.001

**FIGURE 2:**
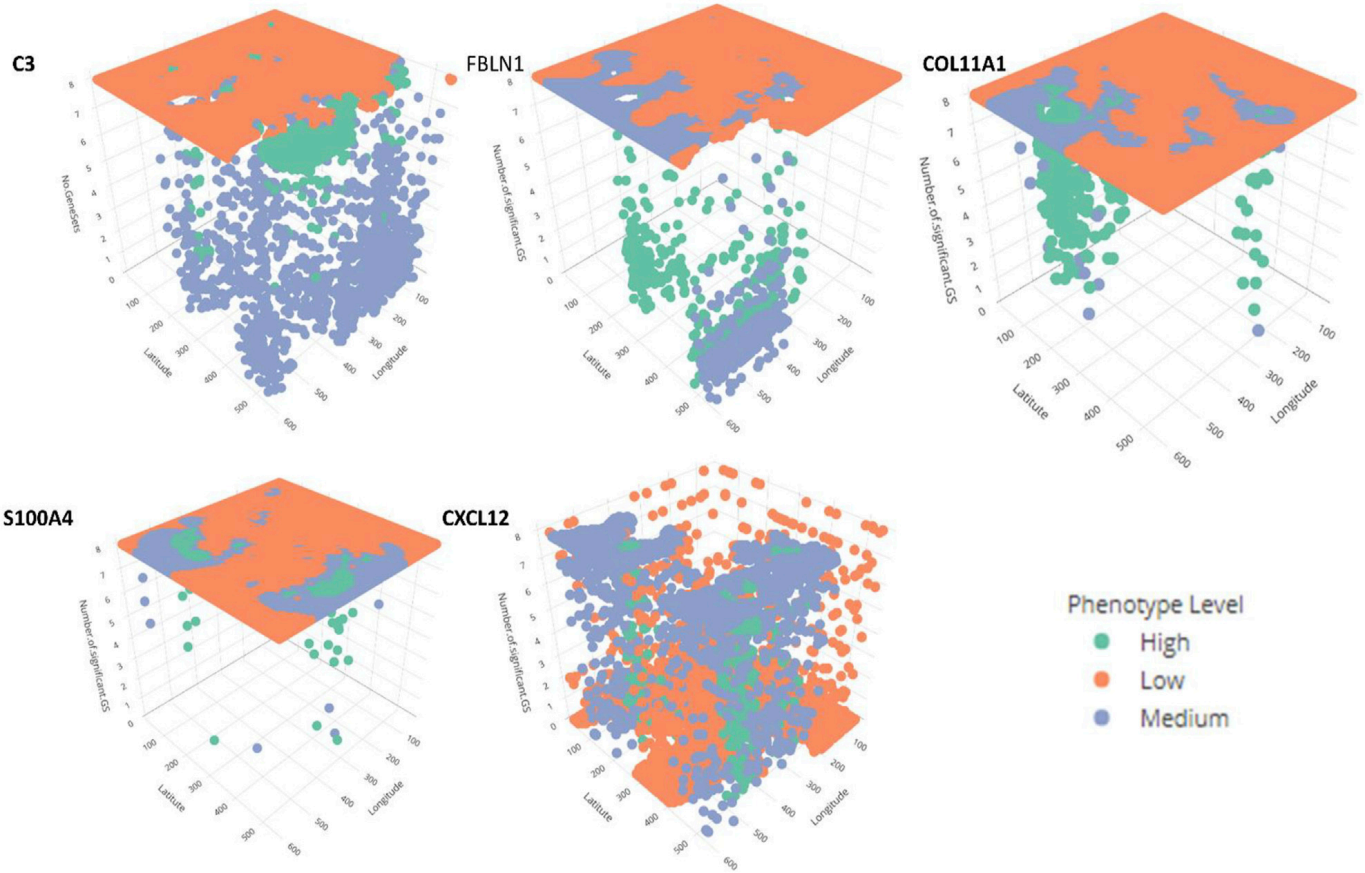
The interactive plots (3D) for the 5 phenotypes at Low, Medium and High CAF levels. Further information about the number and the name of significant gene-sets at each location can be obtained by rotating, zooming, and clicking each dot. The green, orange and blue dots reflect high, low, and medium CAF levels. (This figure is an illustrative static snapshot of the interactive 3D plots accessible online at https://mortezahaji.github.io/GWLCT-Project/.)

**FIGURE 3:**
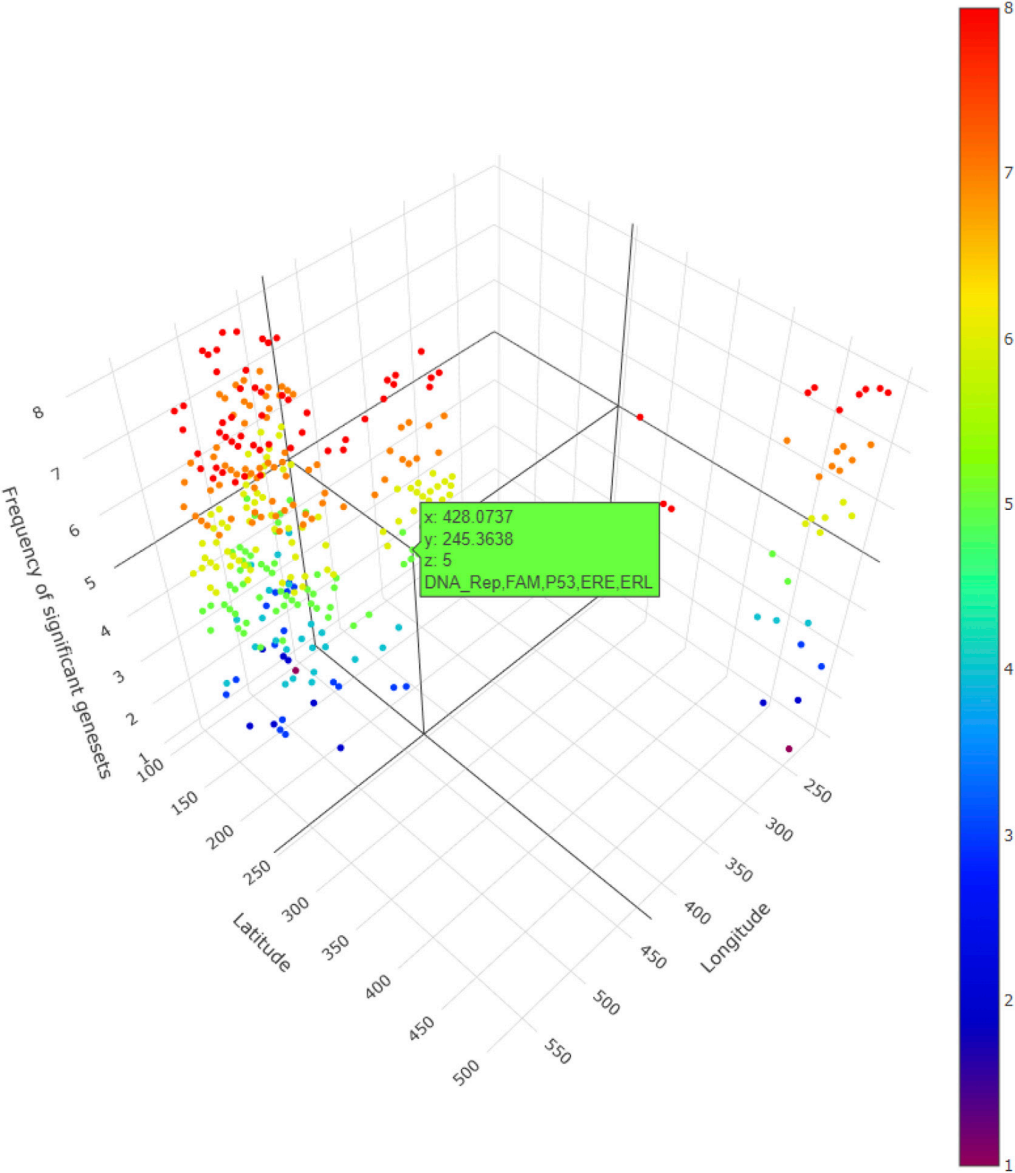
A snapshot of the 3D plot for COL11A1 at high CAF level. The number of significant gene-sets are shown in 8 colors (based on 8 gene-sets). The interactive plots can be accessed at: https://mortezahaji.github.io/GWLCT-Project/.

**FIGURE 4:**
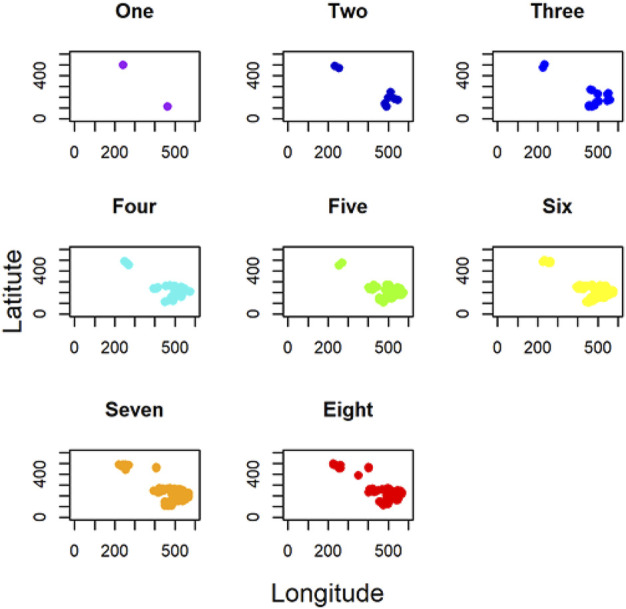
2D plots indicating the number of significant gene-sets across the tumor space for COL11A1 at high CAF level.

In addition, for local GWLCT, three different CAF categories were identified as Low (CAF gene expression less than 0.5), Moderate (CAF gene expression between 0.5 and 1), and High (CAF gene expression exceeding 1). Figure 2 demonstrates a snapshot of the 3D plots for the 5 phenotypes at three CAF levels. A snapshot of the 3D plot for the phenotype COL11A1 at high CAF level is also demonstrated in Figure 3. One is able to detect the frequency as well as the names of significant gene-sets at each location (based on the 8 gene-sets) by clicking on each dot, and rotating and zooming into the 3D interactive plot available at the above-mentioned website. The 3D plot in Figure 3 is divided into eight 2-dimension plots in Figure 4 so that one can evaluate the distribution of one to eight significant gene-sets across the regions with COL11A1 expressed at high CAF level.

**FIGURE 5:**
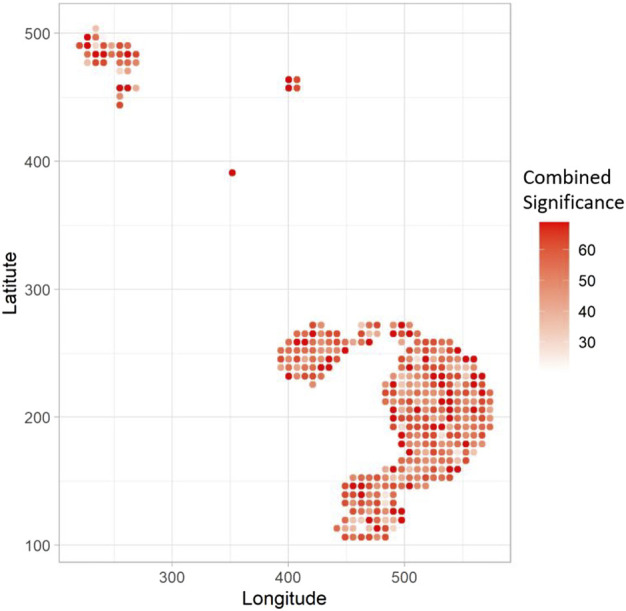
The spatial heatmap shows the Combined Significance (CS) of association of the 8 hallmarks with the COL11A1 phenotype at high CAF level. Bigger the value of CS at a location, the corresponding point is shown in darker red.

Finally, Figure 5 shows the combined significance ( CS ) heatmap of the 8 hallmarks for COL11A1 phenotype at a high CAF level. Higher values of CS in Figure 5 represent locations with a combined significant gene-set.

### Simulation study results

**FIGURE 6:**
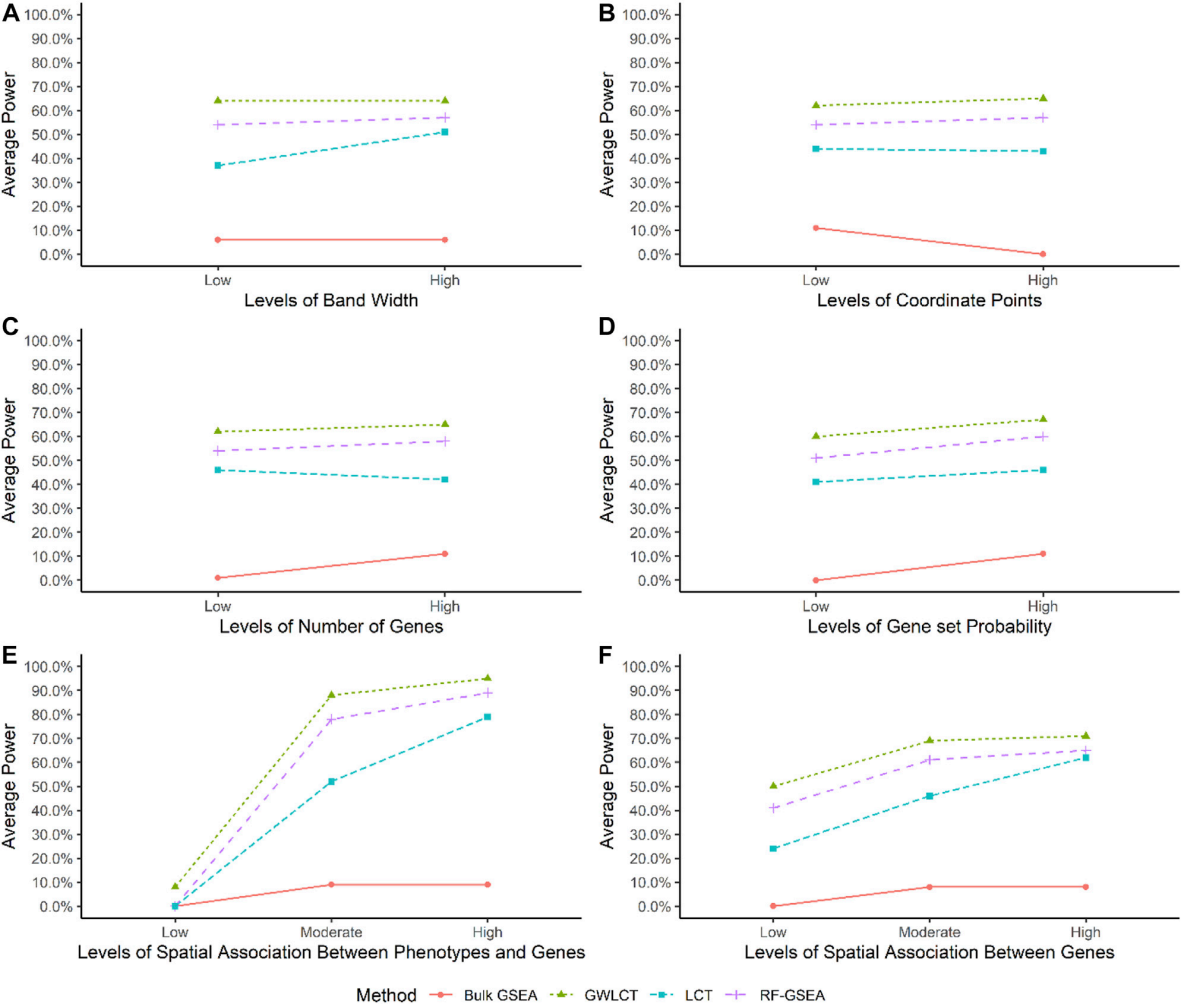
Comparing the average statistical power of each method across various levels of simulation parameters. Considering GWLCT as the reference category and using Dunnett’s test, we compared the average power of GWLCT (green) against Bulk GSEA (red), LCT (blue) and RF-GSEA (purple) by varying from Low to Moderate to High levels of the following parameters: **(A)** the kernel bandwidth, **(B)** the number of coordinate points, **(C)** the number of genes, **(D)** gene-set probability (size of gene-sets), **(E)** the spatial association between genes and continuous phenotype, and **(F)** the spatial association between genes. For detailed interpretation of the results in terms of the testing significance given by the *p*-values, see text.

Supplementary Table S1 and Figure 6 shows the estimated statistical power of the methods using the simulation study. GSEA has the least statistical power among the four methods at all the 144 scenarios. Using 500 iterations in the simulation study, it was revealed that regardless of any parameter in the simulation, GSEA and GWLCT always have the least and most statistical power among the methods, respectively. Overall, we note that bandwidth, number of coordinate points, gene-set size, or total number of genes in each simulation experiment, do not affect the statistical power performance of the methods, a desirable feature shared by sound gene-set analysis methods.

To be particular, by considering GWLCT as the reference category and using Dunnett’s test, we compared the average power of GWLCT against Bulk GSEA, LCT, and RF-GSEA by varying from Low to Moderate to High levels of six different parameters, as shown in Figure 6A through F. (A) For the low bandwidth, the mean power of GWLCT was significantly higher than LCT and Bulk (*p* < 0.001). The GSEA and GWLCT were not statistically different in terms of statistical power (*p* = 0.534). For the high bandwidth level GWLCT was not different with the GSEA (*p* = 0.709), and LCT (*p* = 0.126) and different with Bulk (*p* < 0.001). (B) GWLCT performed better than Bulk (*p* < 0.001 for low and high level of number of coordinate points), and LCT (*p* = 0.0.021 for low level of number of coordinate points, and *p* = 0.002 for high level of number of coordinate points). No statistical difference was found between GWLCT and GSEA (*p* = 0.541 for low level of number of coordinate points, and *p* = 0.715 for a high level of number of coordinate points). (C) GWLCT performed better than Bulk (*p* < 0.001 for low and high levels of number of genes), and LCT (*p* = 0.004 for low level of number of genes, and *p* = 0.019 for high level of number of genes) and the same with GSEA (*p* = 0.599 for low level of number of genes, and *p* = 0.590 for high level of number of genes). (D) GWLCT performed better than Bulk and LCT (*p* < 0.001 for low and high levels of gene-set probability (size of gene-sets)). No statistical difference was found between GWLCT and GSEA (*p* = 0.397 for low level of gene-set probability, and *p* = 0.820 for high level of gene-set probability). (E) For the high level of spatial association between genes and continuous phenotype, GWLCT outperformed Bulk (*p* < 0.001) and LCT (*p* = 0.005). No statistical difference was found between GWLCT and GSEA (*p* = 0.778). For the moderate level of spatial association between genes and continuous phenotype, GWLCT outperformed Bulk (*p* < 0.001) and LCT (*p* < 0.001). No statistical difference was found between GWLCT and GSEA (*p* = 0.399). For the low level of spatial association between genes and continuous phenotype, GWLCT outperformed GSEA (*p* < 0.001), Bulk (*p* < 0.001), and LCT (*p* < 0.001). (F) For the high level of spatial association between genes, GWLCT outperformed Bulk (*p* < 0.001). No statistical difference was found between GWLCT with GSEA (*p* = 0.849) and with LCT (*p* = 0.523). For the moderate level of spatial association between genes, GWLCT outperformed Bulk (*p* < 0.001) and LCT (*p* = 0.012). No statistical difference was found between GWLCT and GSEA (*p* = 0.768). For the low level of spatial association between genes, GWLCT outperformed Bulk (*p* < 0.001), and LCT (*p* < 0.001). No statistical difference was found between GWLCT and GSEA (*p* = 0.528).

The GSEA statistical power is affected by all the simulation variables, however this is mostly due to the zero power of some scenarios. The performance of GSEA is affected by larger number of coordinate points, higher number of genes, higher spatial association among gene expressions, as well as between the phenotype and the gene expressions, and higher number of genes at the gene-sets leads to higher statistical power. Obviously, GSEA is in a lower class of statistical power compared to the other three approaches. Regardless of the scenarios, the results of one-way analysis of variance shows that there are significant differences in the statistical power of LCT, GWLCT, and RF-GSEA (F = 7.842, *p* < 0.001). Tukey’s multiple comparison revealed that the difference is due to lower statistical power of LCT compared to the other two methods. Considering a fixed effect for other variables in the simulation, one can find out that LCT has a lower statistical power compared to the other two GWLCT and RF-GSEA when the band width is low. For a study with a low number of coordinate points, the power of GWLCT is significantly higher in comparison to LCT. The almost flat trend of power for the methods also reveals that the performance of the methods is robust against this parameter. As the number of genes increases, the power of LCT reduces significantly compared to GWLCT and RF-GSEA. Moreover, as the amount of spatial association among genes decreases, the power of LCT is significantly lower compared to GWLCT and RF-GSEA. At high levels of genes spatial association, LCT performs reasonably well compared to GWLCT and RF-GSEA, as it is designed to accommodate correlations across genes in a set or biological pathway, *via* a shrinkage correlation matrix. However, at lower levels of gene spatial correlation, GWLCT and RF-GSEA outperform LCT. Moreover, as the amount of spatial association between phenotype and genes increases, the power of GWLCT is significantly higher compared to LCT and RF-GSEA. In addition, the statistical power is robust against the probability of genes belonging to the gene-sets, which is directly related to gene-set size. Although the power increases slightly for all the methods when the size of gene-sets are larger, the increase is not statistically significant between Low and High levels of binomial probability parameter.

Therefore, the most important variables influencing statistical power are the spatial association features. There is a dose response trend of improvement in statistical power for each method, as spatial association among genes increases. The GWLCT outperforms all other methods in all levels of low, medium, and high spatial association among genes. The performance gap narrows down as we move from Low to High levels, indicating higher magnitudes of correlations are easier to be picked up. Interestingly, GWLCT picks up even on subtle spatial associations across genes, exhibiting the largest improvement in statistical power over other methods at Low spatial association levels. The spatial association between phenotype and gene expressions also plays a key role in method performance. When there is a low spatial association between phenotype and genes, GWLCT is still able to detect true significant associations between gene-sets and phenotype, while all other methods have a flat zero statistical power. Similar to the spatial associations across genes, GWLCT picks up on subtle signals for low levels of phenotype-gene expression levels of spatial associations. GWLCT outperforms all other methods at Moderate and High spatial association between phenotype and genes, with the highest statistical power of 0.95, across all simulation scenarios. A significant increase happens when the spatial association between phenotype and genes increases. The highest power for the GWLCT and RF-GSEA can be achieved when both spatial association variables are high. In contrast to GWLCT, the RF-GSEA loses its statistical power when the spatial association among the genes is at Low levels. GWLCT can identify more subtle signals of spatial association, which is an attractive property of the proposed method. More details on the statistical power of the methods in different circumstances can be found in the appendix Supplementary Table S2.

## Discussion

Observations of heterogeneity of cell subpopulations in a tumor and the complex interplay of functions involved in the diverse morphological and phenotypic profiles of cancers have a long history. Even in the 19th century, pleomorphism of cancer cells within tumors was observed by the “father of modern pathology”, Rudolph Virchow. More recently, in the 1970s, G.H. Heppner, I.J. Fidler and others showed the existence of distinct subpopulations of cancer cells in tumors, which differed in terms of their tumorigenicity, resistance to treatment, and potential to metastasize. Heppner reviewed the concept of tumor heterogeneity in 1984, and recognized cancers as being composed of multiple subpopulations (Heppner and Miller, 1983), which leads to heterogeneity of cellular morphology, gene expression, metabolism, motility, proliferation, etc (Marusyk et al., 2020). Importantly, ITH has been shown to be associated with poor outcome and decreased response to cancer treatment multiple human cancer types implying a general role in therapeutic resistance (Landau et al., 2013; Patel et al., 2014; Zhang et al., 2014).

The past decade has revealed the immense potential of immunotherapy in cancer. Therapies that promote anti-tumor immune responses have resulted in marked and durable responses in subsets of patients in several cancers (Egen et al., 2020). For instance, abundance of tumor-infiltrating lymphocytes (TILs) and absence of lymphovascular invasion were found to be useful prognostic factors for disease-free survival in patients with HR-/HER2+ breast cancer who were treated using adjuvant trastuzumab (Lee et al., 2015). Spatial transcriptomic approach (Ståhl et al., 2016) was used to identify a type I interferon response overlapping with regions of T Cell and macrophage subset co-localization in HER2+ breast tumors (Andersson et al., 2021). To address the complex interplay between different molecular backgrounds that can characterize ITH with spatial precision, we used GWLCT at a given location in the tissue space to test for the association between a phenotype of interest and different selected gene-sets. The CS score to summarize the overall significance of such associations is computed and visualized with a spatial heatmap.

Gene-set analysis (GSA) is a well-established methodological approach in bioinformatics to test for significant regulation of a selected collection of genes across given samples that represent distinct outcomes. At the level of single cells, GSA could be extended to samples that are individual cells which admit to different phenotypes of interest (Dinu et al., 2021). Furthermore, for spatial single cell analysis, such phenotypes would ideally have a spatially correlated and continuous representation. The gene-sets used in GSA are typically curated based on existing experimentally obtained knowledge of genes and their involvements in molecular pathways. In the present study, for illustrative purposes, we selected a collection of 8 gene-sets that represent certain distinctive hallmarks of cancer (Hanahan, 2022). To test for their enrichment in relevant intratumor contexts, we selected 5 different CAF phenotypes of interest since CAFs are well-known for their contribution to heterogeneity and plasticity in the tumor microenvironment (Ping et al., 2021).

The usual methods for GSA involve one of the two major approaches: (a) competitive, which examines if the correlation of a gene-set with the phenotype is the same as the other gene-sets, and (b) self-contained hypothesis, which investigates if the expression of a gene-set changes by the experimental condition. Our LCT method belongs to the former approach which is more likely than traditional methods to detect the regulation of a functional process or biological pathway that is significantly associated with the gene expression results of a given SCA experiment (Dinu et al., 2021). Interestingly, LCT also extends to longitudinal (Khodayari Moez et al., 2019), multivariate and continuous outcomes (Wang et al., 2014), which are capabilities that we built upon here for providing more accurate representation of single cell level stochasticity of the transcriptomic behavior than that of the univariate and discrete class labels typically used in traditional bulk sample studies.

Simulations, along with real omic data analysis, have served as a powerful and effective tool for establishing the performance of new GSA methods. Past studies have thus used simulation for comparative analysis of different criteria of performance of LCT and other major GSA methods. It was found that LCT has type I error and power that are comparable to MANOVA-GSA (Wang et al., 2014) and superior to SAM-GS (Dinu et al., 2007), particularly at higher magnitudes of correlation values across gene-sets (as is commonly noted during GSA). In terms of computational efficiency, LCT outperformed both methods. In another simulation study, LCT also outperformed GSEA (Moez et al., 2018). Along this direction, therefore, in the present study, we conducted a large number of simulations to compare the performance of GWLCT against multiple known GSA techniques based on a variety of well-defined criteria under different experimental assumptions (or scenarios). Interestingly, statistical power did not change with a variation in set size, number of coordination points or bandwidth, or total number of genes in the simulation dataset, for any of the methods considered, which represent desirable properties for sound GSA methods. Larger spatial correlation across gene expression measurements and between genes and phenotype are key aspects of improved statistical power across our simulation experiments.

In the present study, we introduced GWLCT as a new computational platform that presents a fusion of ideas from spatial data analysis (GWR) and bioinformatics (GSA). We understand that the dual modes—both spatial and single-cell—in which GWLCT provides a joint extension to other GSA approaches places it in a unique category thus making it difficult to compare with the existing methods. Yet, we conducted extensive simulation studies which revealed better performance of GWLCT based on several criteria as compared to many known GSA methods that work either on bulk transcriptomics for different scenarios or aspatial version of single-cell transcriptomics. In particular, the use of multiple different kernels and flexible (adaptive) choice of corresponding bandwidths for geographical weighting allows the linear combination test to test for local associations between selected gene-sets and phenotypic contexts within a tumor sample. Thus, GWLCT provides a novel spatial version of gene-set analysis using high-resolution spatial scRNA-seq data. It does have some limitations that will be addressed in our future work. For instance, as it is difficult to determine *a priori* the precise spatial scale at which a gene-set or pathway may be regulated in a given phenotypic context, new multi-scale geographical weighting techniques (Fotheringham et al., 2017) may prove to be useful. We will also extend GWLCT to other omic data as we have previously demonstrated with LCT (Khodayari Moez et al., 2019). As spatial single cell omic platforms become increasingly popular, GWLCT will enrich the ongoing efforts in this rapidly emerging area of research (Zhang et al., 2014; Eng et al., 2019; Hajihosseini et al., 2022). Clinical verification of such new analytical methods will require follow-up studies that must be systematically designed for that specific purpose.

## Data Availability

The de-identified “Spatial Gene Expression Dataset by Space Ranger 1.2.0” used in the present study was obtained from the 10X Genomics website at https://www.10xgenomics.com/resources/datasets/human-breast-cancer-whole-transcriptome-analysis-1-standard-1-2-0 Genomics obtained fresh frozen human Invasive Lobular Carcinoma breast tissue from BioIVT Asterand. It was AJCC/UICC Stage Group I, ER positive, PR positive, HER2 negative. For further details, see the above website.
